# Community health worker knowledge and perceptions of neonatal jaundice in Kumasi, Ghana

**DOI:** 10.21203/rs.3.rs-4662211/v1

**Published:** 2024-07-29

**Authors:** Ann Wolski, Cheryl A. Moyer, Rexford Amoah, Benjamin Otoo, Elizabeth Kaselitz, Ashura Bakari

**Affiliations:** University of Michigan Medical School; University of Michigan Medical School; Ghana Health Service; Ghana Health Service; University of Pittsburgh School of Public Health; Ghana Health Service

**Keywords:** sub-Saharan Africa, newborn, jaundice, community health workers

## Abstract

**Background:**

This study sought to understand community health workers’ (CHW) knowledge and perceptions of community beliefs surrounding neonatal jaundice (NNJ), a treatable but potentially fatal condition prevalent in sub-Saharan Africa.

**Methods:**

In this cross-sectional qualitative study, CHWs in Kumasi, Ghana, completed in-depth interviews with trained research assistants using a semi-structured interview guide. Interviews were audiotaped, transcribed verbatim, and analyzed using grounded theory methodology.

**Results:**

Knowledge of NNJ varied widely among the 23 respondents: 74% knew NNJ could cause death, 57% knew how to screen for NNJ. 35% of CHWs favored home treatment (sunlight therapy or watchful waiting). Three main themes emerged: CHWs perceived that caregivers prefer home treatment, equating hospital care with death; sunlight and herbs are the most common home treatments; and caregivers attribute NNJ to supernatural causes, delaying jaundice diagnosis.

**Interpretation:**

Incomplete understanding of NNJ among trained CHWs and local communities will require improved education among both groups to improve outcomes.

## Introduction

Neonatal jaundice (NNJ) is a common, treatable condition that continues to cause disproportionately high morbidity and mortality in sub-Saharan Africa. It is estimated that as many as 60–80% of newborns worldwide will develop jaundice in their first week of life [1, 2], While many cases are self-limiting, others can progress to hearing loss, brain damage (kernicterus), cerebral palsy, and death [3]. Globally, NNJ is the seventh leading cause of death in the early neonatal period (0–6 days) [4]. However, progression to death or disability can be prevented by proper recognition of jaundice and rapid treatment [5].

Approximately 35% of deaths due to neonatal jaundice occur in Sub-Saharan Africa, with a prevalence of 119/100,000 live births. In comparison, the prevalence in high-income countries (HICs) is 1/100,000 live births [5]. The genetically inherited condition glucose-6-phosphate - which is prevalent in Sub-Saharan African - is a risk factor for the development of severe NNJ [3]. Moreover, infants in Sub-Saharan Africa are more likely to be born at home, or, if born at a hospital, less likely to be screened for conditions predisposing them to severe jaundice [4, 6]. Most infants develop NNJ after 48 hours and hospital discharge [7, 8]. These conditions place the burden of NNJ recognition and care-seeking on infant caretakers and community health workers (CHWs), who are in charge of both maternal education and neonatal home visits. At the same time, we know that most new mothers interviewed at health facilities in Ghana report not knowing the main signs of severe jaundice [3].

It is therefore crucial that caregivers and CHWs are prepared to recognize jaundice and take appropriate next steps. Since Community Health Workers are an important intermediary between facilities and women discharged home after birth (or who give birth at home), this study sought to understand CHW knowledge and perceptions of community beliefs surrounding NNJ.

## Methods

### Study Site

This cross-sectional study was conducted at Suntreso Government Hospital (SGH), in Kumasi, Ghana. Kumasi is the capital city of Ghana’s Ashanti region, and the nation’s second largest city, with a population of 3.3 million. SGH is a district hospital serving Kumasi’s Bantama District, which comprises 52 communities, 32 public and private health facilities, 23 community-based health planning and services (CHPS) zones, and 19 electoral areas.

### Research participants

The community health workers at SGH are responsible for home health visits, family planning, pregnancy care, postnatal outreach, immunizations, growth monitoring, child welfare clinics, and school health services. Of the 25 CHWs working directly with the SGH Public Health Office, 23 were available for interview during the study time period.

### Data collection

Interviews were conducted by two local, trained research assistants (RAs) using a structured interview guide. Each interview was conducted privately, recorded on the interviewer’s iPhone, and transcribed verbatim. Although all interviews were conducted in English, the CHWs occasionally used Twi, the local language in Kumasi; in these cases, the RAs – fluent in Twi – translated the response to English. After interviews, each participant was offered a small gift worth approximately 1USD. Interviews were conducted from April – May 2021, at which point the research team determined that thematic saturation was reached.

### Interview guide

The interview guide used by the RAs was designed to examine community perceptions and behaviors surrounding neonatal jaundice, as part of a larger study assessing the feasibility of a home screening tool for NNJ. The RAs used open-ended and short-answer questions, as well as occasional follow-up probes, to elicit insights into each CHW’s own experiences diagnosing jaundice; her individual community’s beliefs and practices regarding jaundice; and potential barriers to acceptance and use of the home screening tool. The interview questions and responses were reviewed regularly throughout the study, to ensure that unanticipated issues worthy of further inquiry were not being overlooked.

### Analysis

Grounded Theory, a qualitative approach for collecting and analyzing data without imposing previously constructed theoretical frameworks [9, 10] anchored this research. This approach was chosen to capture participants’ perspectives without assuming they would conform to the researchers’ ideas. Open-ended discussions with community health workers were used to generate a preliminary interview guide (rather than anchoring the interview guide in established health behavior frameworks). The preliminary interview guide was then pilot tested and refined to ensure it reflected women’s language and common understanding of neonatal jaundice.

All transcripts were read by at least two members of the research team, and a coding schema was developed to reflect emerging themes. All transcripts were merged into a single Microsoft Word / Google Doc document and coded via highlights and comments. This mechanism was chosen to maximize engagement of research team members without access to NVIVO qualitative software, as the coded document was easily shareable and additional inputs were possible. At the same time, frequencies were tabulated to illustrate the distribution of knowledge and attitudes across respondents. Split frame methodology in the mixed methods tradition [11] was used to compare quantitative and qualitative responses.

### Ethics

All procedures and experimental protocols were reviewed and approved by the ethical review board of the Kwame Nkrumah University of Science and Technology (CHRPE/AP/145/21). The University of Michigan (HUM00198232) exempted the study from ongoing review. All methods were carried out in accordance with relevant guidelines and regulations. All participants were taken through a written informed consent process prior to participation, and consent was obtained from all research subjects.

## Results

Of the 23 respondents, 20 were female (86.96%) and three were male (13%). Their ages ranged from 23–46, with a median of 33. Their official job titles were Community Health Nurse (21, 91%), Public Health Nurse (1, 4%), and Rotation Nurse (1, 4%). Post-qualification experience ranged from three months to 13 years, with a mean of 6.8 years. Further demographic and professional information is detailed in Table 1.

### CHW Knowledge of Neonatal Jaundice

CHW knowledge of neonatal jaundice physiology ranged (Table 2). Twenty CHWs (87%) thought NNJ preventable, with preventive measures including breastfeeding (7, 30.4%), antenatal care (ANC) visits (7, 30.4%), good maternal nutrition (7, 30.4%), early detection of jaundice (4, 17.4%), good hygiene (3, 13%), and education (3, 13%). Risk factors the CHWs identified for jaundice included preterm birth/low birth weight, sickle cell disease, Rh incompatibility, liver disease, formula feeding, and poor maternal diet.

Many CHWs were aware that jaundice is treated with phototherapy (16, 69.6%) and possible blood transfusion (7, 30.4%). Three CHWs (13%) said they did not know how jaundice was treated, and others listed inefficient treatment methods, including antibiotics, medications, and fluid resuscitation. The majority of CHWs (17, 73.9%) knew that NNJ can lead to death. They listed brain and liver damage, growth restriction, and ophthalmologic complaints as other potential outcomes.

When asked about physical exam screening for neonatal jaundice (Table 3), 13 of the CHWs (56%) mentioned both blanching the baby’s skin and checking the baby’s eyes for yellowing; the remaining 10 CHWs (44%) missed these key screening techniques. While 17 CHWs (74%) believed that there was a difference in assessing neonatal jaundice indoors versus outdoors, only 12 (52%) were able to accurately explain the difference. Two CHWs, for instance, said that outdoor sunlight allowed for better skin assessment, but that it was better to check the eyes indoors.

Upon identifying jaundice in a neonate, 15 of the CHWs (65%) said they would refer the child to the hospital immediately. The remainder advised sunlight exposure (6, 26%) or watchful waiting (5, 22%), or they said that their next steps would depend on how severe the jaundice was (4, 17%). For instance, one CHW recommended hospital care for any skin yellowing, but watchful waiting for eye yellowing. Another CHW said,

We can tell if it’s mild or severe. When, after a week, it’s still there, it becomes severe in the forehead and the whole body turns yellow. In that one, we know it’s severe.

All CHWs were interested in learning more about jaundice physiology, diagnosis, and treatment.

### Community Perceptions of Neonatal Jaundice

CHWs reported different community knowledge and management of neonatal jaundice (Table 4). The majority of communities (13, 57%) believed the cause of NNJ to be spiritual, such as ghosts, witchcraft, or the evil eye. As one CHW said,

Most of them [the mothers] think it’s a curse, like someone has bought it for them. It’s a spiritual something. That is what most of them think. When they give birth, they don’t want to come out. They want to stay indoors for some time, so that no one will see their baby. If someone sees their baby, they might give something…bad.

Other communities attributed NNJ to an oily maternal diet (5, 22%), or to poor maternal health and lack of ANC attendance (5, 22%). Most communities (12, 52%) were split between home and hospital management of jaundice. Six communities (26%) relied exclusively on hospital management, and five communities (22%) relied exclusively on home management. The most popular method of home management was sun exposure (15, 65%), but multiple CHWs also mentioned herbal supplements, breastmilk, enemas, and herbal baths as home regimens.

Several CHWs noted that caregivers in their communities preferred home management to hospital care, until jaundice became severe and potentially irreversible. They reported,

They will just sit at home for it to get deteriorated before they are brought to the hospital.

Yeah, they know everything needs to be treated at the hospital. They know it. But they will stay at home and do home remedies – herbal drugs and all those things. But when it gets to the later part, when you can’t do anything about it, that’s when they think, let me send the baby to the hospital.

Five CHWs also noted that most mothers equate hospitals with death, and avoid taking their babies there out of fear:

“The moment [I refer to the hospital], they start crying; they won’t go. They will not go at all, because they think that place is for serious conditions. So the moment you are referring her to them, then their baby’s going to die – they start crying. .. No matter what, she will not go. So when we look at such a mother, we try to manage at our level [of healthcare facility]. Because they don’t like going to the higher level. They think when they go there their baby will die. So they prefer the CHPS compound, the health center – [hospitals], they won’t go.”

“When you refer them to the hospital, they think when they go they will not come back - they will die. They have that superstition. So when you refer them, they will accept the letter, but they will not go.”

## Discussion

This study demonstrated incomplete understanding of NNJ, its diagnosis, and its treatment, among both trained CHWs and local communities, as perceived by CHWs. Among CHWs, 74% knew that NNJ could cause neonatal death. However, only 57% knew how to appropriately screen for NNJ, and 35% favored home treatment for jaundice, in the form of either sunlight therapy or watchful waiting. Regarding CHW understanding of community perceptions of NNJ, several themes emerged: most caregivers prefer to treat jaundice at home, equating hospital care with death; the most common home treatments are sunlight and herbs, followed by increased breastfeeding, enemas, and baths; and many caregivers attribute NNJ to supernatural causes. One notable finding was the repeated mention of the fear of the “evil eye,” causing mothers to keep their babies indoors and wrapped in blankets, in turn complicating or delaying jaundice diagnosis.

The majority of previous studies assessing CHW knowledge of NNJ in Africa have taken place in Nigeria [12, 13, 14, 15]. Nevertheless, these studies revealed similar shortcomings in CHW diagnosis and treatment of jaundice, with only 35.5–75.8% demonstrating proper exam techniques and 58–74.2% referring NNJ cases to the hospital [12, 15]. More CHWs in Nigeria recommended inappropriate glucose water (67.1%) or natural phototherapy (66.7%) treatments for jaundice [15]. However, Nigerian CHWs had greater knowledge of NNJ complications, with 88.5–100% identifying death as a possible outcome.

Our study, conducted in the Ashanti Region of Ghana, provides a valuable complement to the limited existing research in Ghana related to NNJ understanding amongst expentant mothers. Seneadza et al (2022) explored knowledge, attitudes and perceptions among mothers attending antenatal and postnatal clinics in the southern and eastern areas of the country [3]. Despite being conducted at least in part in the capital city (Accra), where rates of maternal education are the highest in the country, women’s knowledge of neonatal jaundice was extremely variable [3]. Similarly, a study conducted in 2013 at two healthcare facilities in Accra found that only 5% of expectant mothers attending antenatal clinics could identify one or more correct methods of NNJ treatment, and less than half could identify danger signs [16]. This suggests a persistent deficiency in community-health education related to NNJ in Ghana, despite its prevalence within the population.

Our study had numerous strengths, including open-ended questions to elicit CHW ideas and perspectives, and thematic saturation of the CHWs serving a large catchment area in an urban center. One limitation was that our assessment of community practice was focused on perceptions of CHWs, and not on interaction with primary sources. Additionally, the interviews took place during early 2021, in the middle of the SARS-CoV-2 pandemic. While the COVID-19 impact in Ghana was significantly smaller than many other places (with less than 100,000 total cases by the time of this study, in a country of nearly 30 million), it is possible that CHW perceptions were swayed by pandemic changes in behavior [17].

Nevertheless, this study has several important implications. CHWs play an important role in caregiver education and neonatal assessment, but currently possess inadequate knowledge to assess and treat jaundice. Of particular salience is the lack of understanding of the importance of proper lighting to assess for NNJ, as well as the importance of hospital referral for all cases of suspected jaundice. Communities too displayed knowledge gaps surrounding NNJ. The commonly held fears of witchcraft, outside exposure, and hospital treatment of neonates combine to reduce the likelihood of early jaundice identification and treatment, in turn increasing morbidity and mortality from this treatable condition. Given that CHWs are a primary source of education and medical assessment for many neonatal caregivers, we hypothesize that improved CHW education around NNJ will benefit communities many-fold.

Reductions in NNJ morbidity and mortality will require improved education among both CHWs and community members, with a focus on newborn caregivers. Further research is needed into the most effective educational modalities for each group, to ensure rapid NNJ diagnosis and treatment.

## Figures and Tables

**Table 1:** CHW Demographics and Professional Statistics

CHW Characteristics		Number	Percent	Mean	Median
Sex (n = 23)	Female	20	87.0%		
	Male	3	13.0%		
Age (n = 23)				32.9	33
Position (n = 23)	CHN	21	91.3%		
	Public health nurse	1	4.4%		
	Rotation nurse	1	4.4%		
Sees babies at: (n = 23)	Home visits	20	87.0%		
	CHPS	16	69.6%		
	Hospital	12	52.2%		
	Other	1	4.4%		
Has seen baby with jaundice (n = 23)	Yes	20	87.0%		
	No	3	13.0%		
Years of post- qualification experience (n = 23)	<1	1	4.4%	6.8	8
	1–4	7	30.4%		
	5–9	9	39.1%		
	10+	6	26.1%		
Years at current site (n = 23)	<1	4	17.4%	5.0	5
	1–4	6	26.1%		
	5–9	12	52.2%		
	10+	1	4.4%		
Babies seen per month (n = 20)	<50	5	25%	167	100
	50–200	10	50%		
	>200	5	25%		
Jaundiced babies seen per month (n = 20)	0	8	40%	1.4	0.75
	1–2	8	40%		
	3 +	4	20%		

**Table 2:** CHW Knowledge of Jaundice Physiology

Question	Responses	Number	Percent
Can we prevent jaundice? (n = 23)	Yes	20	87.0%
	No	1	4.4%
	Don’t know	2	8.7%
How can we prevent jaundice? (n = 23)	Breastfeeding	7	30.4%
	ANC visits	7	30.4%
	Proper nutrition for mother	7	30.4%
	Early detection and treatment	4	17.4%
	Infection prevention/hygiene	3	13.0%
	Education	3	13.0%
What puts babies at higher risk of developing NNJ? (n = 22)	Preterm birth/LBW	10	43.5%
	Sickle cell disease	4	17.4%
	Rh incompatibility	2	8.7%
	Liver disease	2	8.7%
	Formula feed	2	8.7%
	Poor maternal diet	2	8.7%
What kind of treatment are babies with NNJ given at the hospital? (n = 23)	Phototherapy	16	69.6%
	Blood transfusion	7	30.4%
	Unknown medication	6	26.1%
	Antibiotics	3	13.0%
	Fluid resuscitation	3	13.0%
	Breast milk	2	8.7%
	Don’t know	3	13.0%
What are the consequences if NNJ is not treated? (n = 23)	Death	17	73.9%
	Brain damage	7	30.4%
	Liver damage	7	30.4%
	Growth restriction	6	26.1%
	Eye damage/blindness	5	21.7%
	Cerebral palsy	2	8.7%

**Table 3:** CHW Assessment and Management of Jaundice

Question	Responses	Number	Percent	Salient Quotes
Does the CHW have an accurate screening method for NNJ? (n = 23)	Yes (mentions checking eyes and blanching skin)	13	56.5%	
	Yes, with error (does not check eyes or does not blanch skin)	10	43.5%	
Is there a difference in assessing babies for NNJ indoors vs. outdoors? (n = 23)	Yes	17	73.9%	“There may not be light in the room. So you cannot see the baby’s eye very well. You have to come outside and check it.” “If you are okay with it, I will do it indoors, where you are comfortable.”
	No	6	26.1%	
Is the CHW able to explain the difference between indoor and outdoor assessment for NNJ? (n = 23)	Yes, clearly	12	52.2%	“When the baby is inside, you will not see that the person has jaundice, because of the darkness of the room. But if you are outside, because of the sun and the light, you can really see it.” “What we do is in the room, with their eyes - that is when the babies are sleeping. You can easily detect it from the whiter part of their eye. With their skin, that’s outside.” “Physical examination will be done inside, because. .. The child needs privacy. If they have abnormality, their parent does not want the other person to know. For them, we provide privacy.”
	Yes, with error	5	21.7%	
	No	6	26.1%	
What do you advise when you see a baby with NNJ? (n = 23)	Report to hospital immediately	15	65.2%	“If I check and it’s not severe, and I think it’s mild, then I tell the mother to continue to give her breastmilk.” “Maybe after exposing the baby for about three to four days to the sun, when the sun rises, and continuous breastfeeding, every thirty minutes you breastfeed the child, and still the condition is like that - the yellowish is there, doesn’t change, the tongue is yellow, the feet, everything - nothing has changed. And the baby cannot suckle well. And the baby is a little bit weak. They can’t be moving their limbs like the normal babies. You can see that there’s no improvement - it’s becoming severe. So we refer it.”
	Expose baby to sunlight	6	26.1%	
	Watchful waiting	5	21.7%	
	Depends on level of NNJ	4	17.4%	
What makes you refer a baby with NNJ to the hospital? (n = 23)	Color only	13	56.5%	“If I see any sign [of jaundice] on that baby, I tell the mother to immediately bring the baby to the facility.” “[The danger signs are] difficulty in breathing. And also if she can’t suckle very well. Sometimes she will be really uncomfortable.” “When the skin is turning yellow, that is when we refer. If it’s only the eyes, after three days it’s only seen in the yes, then we advise on exclusive breastfeeding. But when you can see it on the feet or the tummy or the palm, that’s very alarming. They have to send the baby quick for treatment.” “If after a weekend, three to four days, it’s still there, I will refer the mother to come to the hospital.”
	Danger signs	4	17.4%	
	Location/intensity of jaundice	4	17.4%	
	Lack of improvement/worsening	4	17.4%	

**Table 4:** Community Knowledge and Management of Neonatal Jaundice

Question	Response	Number	Percent
What do people in your community believe causes jaundice? (n = 23)	Spiritual causes/evil eye	13	56.5%
	Oily maternal diet	5	21.7%
	Lack of ANC/poor maternal health	5	21.7%
	Don’t know	2	8.7%
How do people in your community manage NNJ? (n = 23)	At home	5	21.7%
	In the hospital	6	26.1%
	Split between home/hospital	12	52.2%
How do people in your community treat NNJ at home? (n = 23)	Sunlight	15	65.2%
	Herbs	10	43.5%
	Breastmilk	4	17.4%
	Enema	3	13.0%
	Baths	3	13.0%

## Data Availability

Data are available from the authors upon reasonable request.
